# Assessment of digital light processing (DLP) projector stimulators for visual electrophysiology

**DOI:** 10.1007/s10633-022-09917-4

**Published:** 2023-01-08

**Authors:** Oliver R. Marmoy, Dorothy A. Thompson

**Affiliations:** 1 Clinical and Academic Department of Ophthalmology, Great Ormond Street Hospital for Children NHS Foundation Trust, Great Ormond Street, London, WC1N 3AJ England, UK; 2 Developmental Biology and Cancer Research and Teaching Department, Great Ormond Street Institute for Child Health, University College London, London, WC1N 1EH UK; 3 Department of Life Sciences, Manchester Metropolitan University, Manchester, M15 6BH UK

**Keywords:** Visual evoked potential, Pattern electroretinogram, Stimulator, Monitor, Visual display unit

## Abstract

**Introduction:**

Visual electrophysiology tests require the use of precise and calibrated visual display units (VDUs). Existing VDUs for presenting structured stimuli are now mostly obsolete, with modern solutions limited or unsuitable for clinical testing. Digital light processing (DLP) laser projectors have recently become commercially available and this study aimed to assess their suitability as VDUs for visual electrophysiology testing.

**Methods:**

This study consisted of two sections. The first was a photometric study of two DLP laser projectors (Viewsonic LS831WU and HiSense 100L5FTUK) to assess luminance, contrast, spectral and temporal characteristics of the stimulus. The second was a physiological study comparing pattern electroretinograms (PERG) and visual evoked potentials (PVEPs) amplitudes and peak-times recorded using a DLP laser projector, photometrically and spatially matched to existing plasma VDUs at our institution (Pioneer Electronics Corporation, PDP422MXE).

**Results:**

The Viewsonic DLP laser projector was capable of high luminance levels (0–587.5 cd/m^2^) whilst maintaining contrast above 93%. The temporal properties showed fast rise and fall times of 0.5–1 ms and 0.5–1 ms, respectively, without any transient luminance change with reversals. The device required a warm-up time of at least 2 min until reaching near maximal luminance. The second (Hisense) device was observed to have a detrimental input lag jitter so was not used for any further analysis. PERGs and PVEPs showed high agreement and correlation (*r* = 0.766–0.905) between the Viewsonic DLP device and existing plasma VDUs. No significant differences were observed for P50 and P100 peak-time (*p* =  > 0.05), however P50, N95 and P100 amplitudes were all significantly larger for the DLP device (*p* =  < 0.05).

**Discussion:**

The DLP laser projector tested in this study is a viable and practical replacement VDU for clinical electrophysiology tests of vision. The device is easily capable of meeting ISCEV standards, and showed PERG and PVEP amplitudes larger than existing systems despite photometric and spatial matching. The DLP laser projectors are capable of very large field sizes so are beneficial for paediatric testing or those wishing to examine large field responses. Importantly, it was observed that some devices may suffer input lag jitter, therefore, individual calibration and assessment of DLP projection systems is an important consideration before clinical implementation.

**Supplementary Information:**

The online version contains supplementary material available at 10.1007/s10633-022-09917-4.

## Introduction

Visual display units (VDUs) are essential devices in visual electrophysiology for presenting structured visual stimuli. Typically VDUs generate patterned or multifocal stimuli for clinical visual evoked potential (VEP), pattern electoretinogram (PERG) or multifocal electroretinogram (mfERG) recordings, for which there are international standards [1–3]. There are prescribed technical requirements of such VDUs to ensure that they have sufficient properties of luminance, contrast, colour, alongside temporal characteristics. These precise measurements ensure that the recorded physiological potentials are predictable and reproducible.

There is currently a widespread deficit of adequate commercially available VDUs. Many widely used stimulators are either obsolete, or those available are unsuitable for visual electrophysiology testing. For example, many centres use cathode ray tube (CRT) stimulators despite these being obsolete and parts no longer manufactured, with older models requiring frequent calibration. At the authors institution, plasma display panels (PDP) are used, but are similarly rarely produced and obsolete. Modern VDUs such as liquid crystal display (LCD) screens or organic light emitting diode (OLED) displays can be largely unsuitable for electrophysiology testing. LCD displays unfortunately succumb to a detrimental transient luminance artefact with each pattern element shift to on- or off-states, which in some circumstances can be minimised using low contrast or in-built luminance adjustments, but often are not adequate for testing and risk being non-compliant with ISCEV standards [4–6]. OLED displays are potential solutions to this issue, however many suffer a detrimental input lag jitter due to resampling of the incoming trigger so risk desynchronisation of the recorded response.

Digital light processing (DLP) laser projectors were first developed for the defence industry before being widely used within digital cinema [7]. Developments in technology now mean that these devices are commercially available for personal use and can be used within an ultra-short throw ratio so do not require the large projection distances originally needed for large field sizes. DLP laser projectors involve projection of a light source, the laser, onto a digital micromirror device (DMD). The DMD is comprised of thousands of tiny micromirrors which can be individually controlled into on- or off-states at a rapid rate [7]. Each mirror on the DMD represents a pixel, whereby each mirror reflects the light onto a light absorber or toward a projection lens. The light is typically passed through a high speed colour wheel to achieve a vast range of chromaticity followed by optical correction for the subsequent projector screen. The resultant screen can have very high resolution, luminance, temporal refresh rate and appreciable field size, making it a candidate to replace obsolete VDUs in visual electrophysiology.

The purpose of this study was to assess DLP laser projectors for their suitability for pattern visual electrophysiology tests, both through photometric and physiological perspectives.

## Methods

This study comprised of two major sections. The first stage was based on stimulus calibration and properties of two individual DLP laser projection systems. The second stage was comparison of PVEPs and PERGs from a group seven of healthy subjects from a DLP laser projector against PDP stimulators already established within our centre.

Two DLP laser projectors were assessed within this study (HiSense model 100L5FTUK and Viewsonic model LS831WU). Both devices had an ultra-short throw projection ratio meaning < 30 cm distance was required from the device to projection screen. These were driven from an Espion Diagnosys (E^3^ system) via an integrated graphics processor outputting a 60 Hz signal via a Video Graphics Array (VGA) connection. DLP devices projected onto a white triple-ply fiberglass laminate projection screen with black backing (Sapphire AV Manufacturing Ltd., model SEWS240RWSF-ATR).

### *Section 1—Photometric measurements*

Photometry measurements were made using an ILT1700 photometer and SED033 barrel with 9.27 mm aperture to block ambient light, providing a large candela measurement range, with a Y photopic correction filter. A checkerboard pattern was created to subtend 30 degrees of visual angle at a viewing distance of 125 cm. Measurements were made at 1 cm distance from the image projected onto a large white projection screen following a 10 min warm-up time. Additional measurements were made during the warm-up period, from turn on and immediate display of a checkerboard pattern, to assess warm-up time requirements directly. A spot photometer (Konica Minolta, model LS-110) was used to measure the individual white check luminance distribution across the 30 degree field. This was performed by displaying check widths subtending 2.5° within the 30 degree field and measured sequentially when checks were white. Mean luminance was calculated alongside contrast using Michelson contrast formula ((L^max^—L^min^)/(L^max^ + L^min^)). The temporal characteristics of a reversing (2.3rev/sec) and onset-offset checkerboard stimulus were measured using a photodiode (Hamamatsu electronics, model S1223). The waveforms were assessed for their profile and scrutinised for response time, rise time, fall time and any transient luminance change, which was additionally assessed using a blank white sheet of paper in front of the stimulus screen to visualise any diffused transient luminance changes [8].

Spectral measurements of white checks on the projection screen were made using an ILT960 spectroradiometer. Results were made in continuous and time-integrated mode (< 4 ms sample) to assess whether mean and transient luminance may alter, particularly due to known ‘colour wheel/rainbow effect’ within DLP laser projection systems [9].

Spatial properties were modified and manual measurements of stimulus field size, element size and viewing distance calculated and optimised to replicate spatial characteristics of PDP stimulator systems established within our unit (Pioneer Electronics Corp., model PDP422MXE).

### *Section 2—Physiological measurements*

Seven healthy participants (5 female, age range 27–42 years) were recruited from the staff population at the authors institution and tested using an existing laboratory PDP VDU and the ViewSonic DLP laser projector. No participants had any history of ophthalmic or neurological disease apart from refractive error which was optically corrected during testing. Photometric properties of the ViewSonic DLP laser projector were matched to the existing PDP device using the device settings.

All procedures performed were in accordance with institutional standards (approval ref. 3352) and with the 1964 Helsinki Declaration and its later amendments or comparable ethical standards.

PVEPs were recorded using a single-channel occipital electrode (Oz) referred to a mid-frontal reference (Fz) with ground electrode placed centrally (Cz). Electrode impedances were maintained below 5kΩ. PERGs were recorded with a corneal fibre electrode referred to an electrode placed laterally to the outer canthus. A range of high contrast black and white check widths (Michelson contrast ≈ 96%), were presented ranging from 200’-6’ in a large field (30°) binocuarly in random order. Luminance measurements were matched to existing PDP devices as per Sect.  1. PVEPs and PERGs were recorded simultaneously to each stimulus with a reversal rate of 3.15/sec. Recordings for both VDU devices were made for each participant within the same session with the same electrodes, to minimise variability. Resultant signals were amplified and sampled at ~ 4000 Hz, with a minimum of 100 sweeps obtained per average and a minimum of two averages taken per check width. Filter settings were 0.3–300 Hz.

Resultant signals were measured in terms of N75-P100 trough to peak amplitude and P100 peak-time for PVEPs, and P50 amplitude and peak-time and N95 amplitude for both devices. The N95 peak-time was not used as is this is often broad and variable and not often used clinically [2].

Amplitudes and peak-times of respective PERG and PVEP components recorded from each device were plotted on scatter plots and two-tailed Pearson correlation coefficient calculated to visualise and to assess the relationship between devices. Amplitude and peak-times produced to each check width were then stacked between devices and Bland–Altman plots performed for all components to assess limits of agreement. PERGs for 6’ were not reliably evident for all participants or low amplitude for others so were not included in analysis. These data were assessed for significant differences using a paired sample *t*-test or Wilcoxon signed-rank test depending on their normality.

## Results

### *Section 1—Photometric measurements*

Both devices were capable of displaying patterned stimuli across a very large fields (up to 100 inches/254 cm diagonally) from the Espion E^3^ system. It became evident in early testing using the photodiode that the Hisense DLP device was detrimentally affected by an input lag jitter (supplemental Fig. 1). Despite modification of synchronisation speeds and input frame frequency (60–120 Hz), the resultant signals shifted unpredictably between 0 and 30 ms, and it was not possible to time-lock the electrographic signals accurately. Accordingly, no further analysis took place for the Hisense DLP laser projector. Fortunately, the Viewsonic DLP laser projector did not experience this issue and was used for all subsequent analysis and tests.

**Fig. 1:**
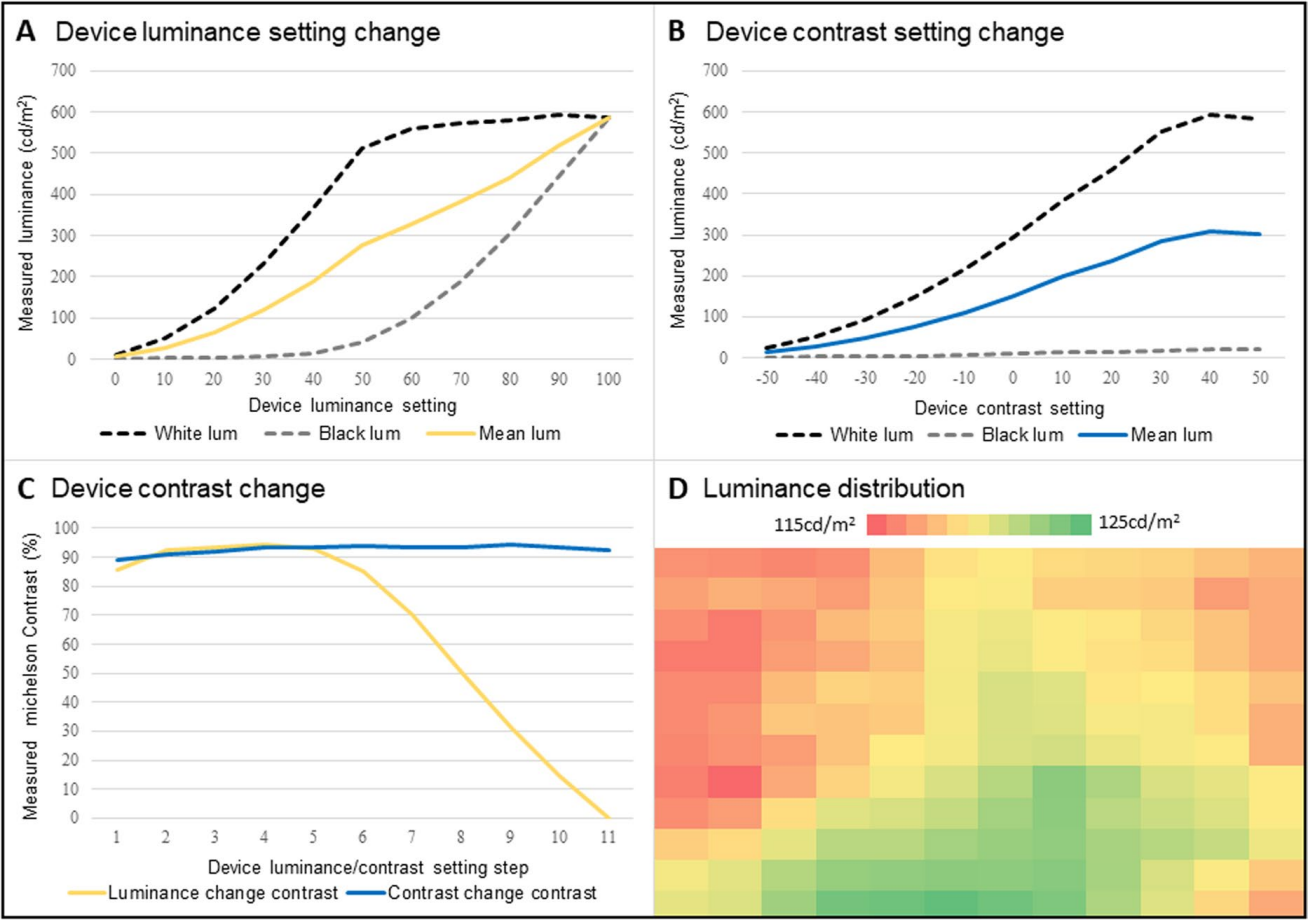
Luminance and contrast properties of DLP laser projector stimuli. Panel A demonstrates the effect of increasing the device luminance setting on the luminance of white (black-dashed line), black (grey-dashed line) and mean (yellow solid line) luminance. Panel B demonstrates the effect of increasing the device contrast setting on white (black-dashed line), black (grey-dashed line) and mean (blue solid line) luminance. Panel C demonstrates that above a device software luminance setting of 30, the contrast reduction falls to unacceptable levels (yellow solid line), whereas at a set device luminance but contrast setting change, the contrast is maintained above 90% (blue solid line) with increasing luminance seen in panel B. Panel D shows the luminance distribution of white checks across a 30 × 30° field, each square represents a 2.5° white check luminance. This was the measured luminance matched to the existing laboratory PDP device, with minimum luminance (115 cd/m^2^ as red and 125 cd/m^2^ as green)

Device settings were modified to alter luminance and contrast, respectively (Fig. 1A–C). The Viewsonic DLP laser projector was capable of very high luminance levels, with white checks measuring up to 594.9 cd/m^2^ (Fig. 1A–B). It was found that maintaining luminance (device software setting of 30) and altering contrast, maintained contrast above 90% for a range of mean luminance levels up to 302.2 cd/m^2^. Whilst contrast was maintained above 93% for mean luminances up to 100 cd/m^2^ (Fig. 1C), at high device luminance settings black checks became brighter between 1.4 and 22.2 cd/m^2^ (Fig. 1A). No specific reference is made to black check luminance in the ISCEV standard other than the relative contrast must remain high, although one must consider that dark checks are ideally a minimally stimulated area and increasing this may affect reproducibility and theoretically affect clinical applications. The measured luminance of white checks across the visual field ranged from 114.8 to 125 cd/m^2^, with the maximum deviation from maximal luminance being 8.2%. This tended to show a spatial distribution with maximal luminance in the inferior central portion of the field and minimal luminance in superior areas laterally (Fig. 1D).

#### Spectral measurements

**Fig. 2:**
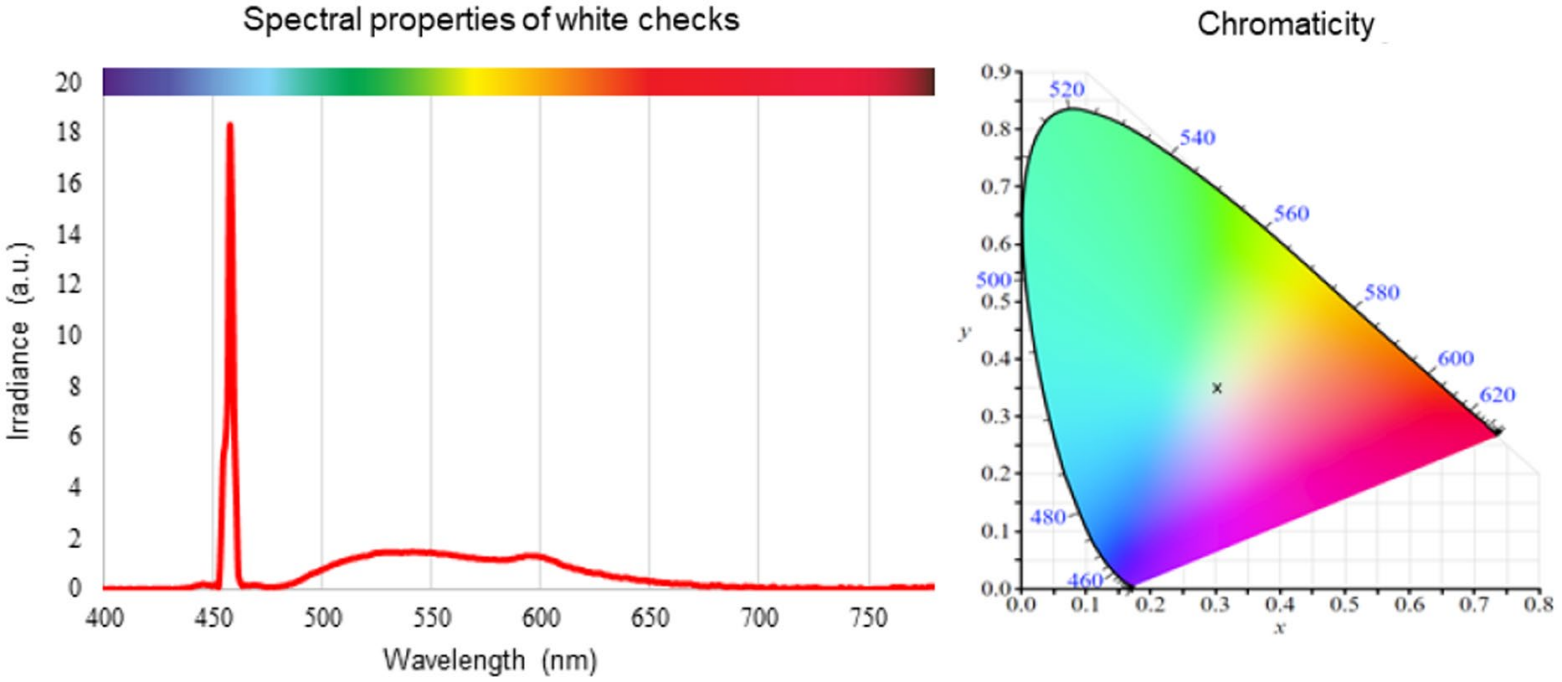
Spectral properties of the Viewsonic DLP laser projector measured from white checks. The left panel shows the relative irradiance of a white check over a wavelength spectra. This shows a large peak in the blue range (457 nm) with broad profile between 470 and 700 nm with broad peaks at 542 nm and 595 nm, respectively. The right panel shows the chromaticity diagram with the DLP laser projector white check CIE coordinates at *x* = 0.310 and *y* = 0.349 (CIE 1931 colour space) seen as the black cross, with a correlated colour temperature of 6495K

Spectral measurements of the white checks demonstrated that the spectral profile of the DLP laser projection system has a large peak within the ‘blue’ range (457 nm), with subsequent broad spectra between 470 and 700 nm, respectively, with subtle broader peaks at 542 nm and 595 nm, respectively (Fig. 2). The CIE (1931) coordinates of the stimulus were *x* = 0.310 and *y* = 0.349, with a correlated colour temperature of 6495K. One additional observation during these assessments was with fast eye movements (i.e. rapid saccades), one could observe a stroboscopic ‘rainbow effect’ (supplementary Fig. 2).

#### Warm-up times

One consideration we took for assessing luminance of VDUs was warm-up times, which reflects the time a device takes once turned on to reach a constant and stable luminance ready for testing. We assessed the warm-up time of the DLP laser projector and PDP systems based at the unit, measuring how long each device took to reach their maximum luminance of a checkerboard (Fig. 2).

**Fig. 3:**
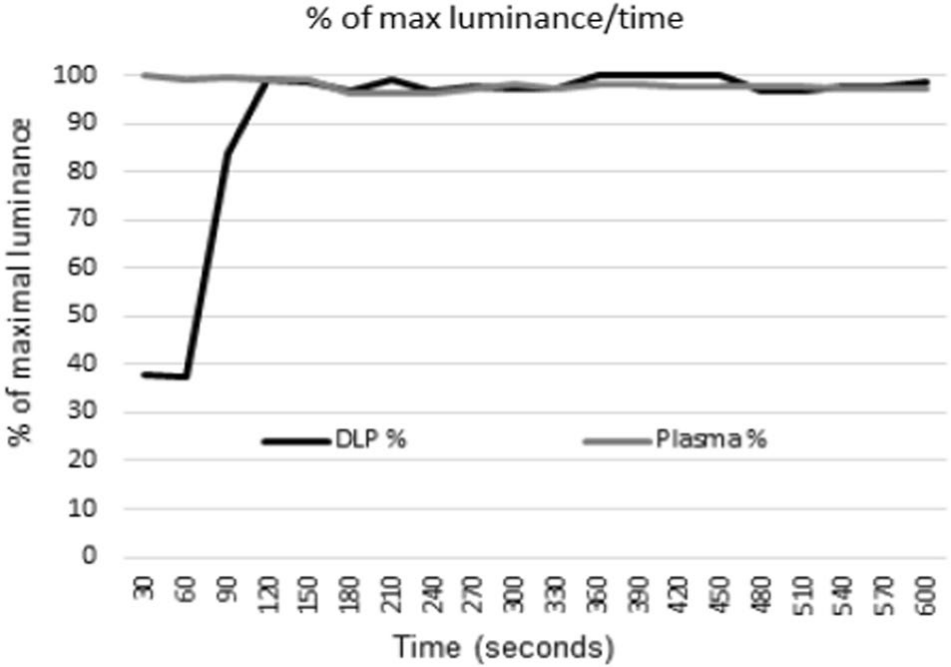
Visual display unit (VDU) warm-up times. Each VDU is plotted according to their % of maximal luminance (Y axis) against time (x-axis)

We found that PDP devices require no warm-up time, whereas the DLP device took around 2 min to reach > 95% luminance thereafter remaining stable. Response time of the checkerboard reversal was also recorded for 10 min from turning on and was stable throughout this period of measurement (Fig. 3).

#### Temporal profile

**Fig. 4:**
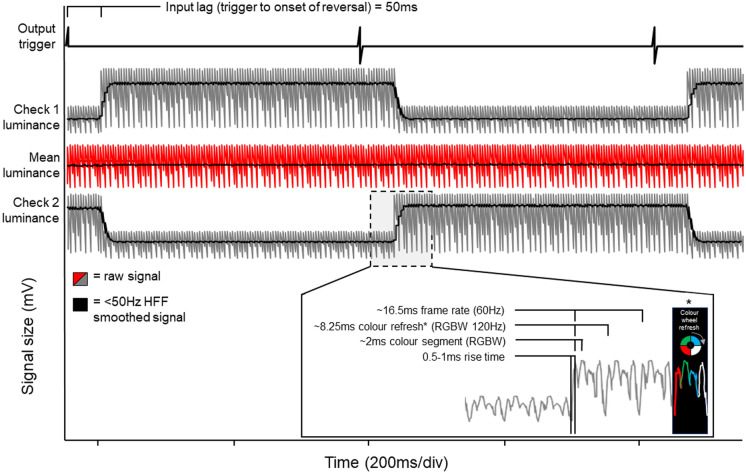
Temporal profile of the DLP laser projector stimulus used in this study. A 2.3 rev/sec square-wave checkerboard pattern was presented. Two photodiodes were placed on the screen, one on a white check ‘Check 1’ and one on a black check ‘check 2’ before starting. A minimum 100 responses were averaged. The top black signal is the output trigger, showing a 50 ms time period until the onset of the check 1 luminance (input lag). The mean luminance (check 1–check 2) was stable throughout the recording. The black superimposed signal on check luminance signals are those that are the same signal with a high frequency filter (0–50 Hz filters) to show the gross morphology of waveforms—importantly the rise and fall times were not measured from the filtered signal as this would produce false values. The zoomed signal shows the frequency components and rise times, corresponding to frame rate, colour refresh and colour segment, respectively. An illustrative example of each colour segment piece is seen within the black box explaining the waveform morphology

The temporal profile of the reversing checkerboard pattern signal was very fast with the DLP laser projector. The measured rise and fall times were equal and were 0.5–1 ms in duration (Fig. 4). A constant luminance was evident for the duration of the reversal phase. The signal from the photodiode, in addition to the 2.3rev/sec reversal frequency, also comprised of three fundamental high frequency components of 60 Hz, 120 Hz and ~ 480 Hz. These correspond to the output frame rate from the Espion system, colour wheel frequency (× 2 of framerate) and individual colour wheel segment changes, respectively (Fig. 4 zoomed panel). No noise associated with mains electrical frequency (50 Hz) was observed. The input lag (i.e. the time taken from signal output to onset of a stimulus change) was fixed at 50 ms with no jitter. Importantly, no transient changes in mean luminance for reversal or onset-offset stimulation were seen.

#### Spatial properties

The Viewsonic DLP laser projector is capable of very large field sizes, outputting to a 100 inch (254 cm) screen in 4 k resolution, which would equate to 90 degree field at our working distance of 125 cm. Whilst very large field stimulation may be advantageous in some circumstances, we replicated a field size subtending 30 degrees to match the existing PDP stimulators at our centre and to minimise phase cancellation from large paramacular PVEP components [10]. The DLP device was capable of projecting a range of check widths, and maintained high resolution for small check widths (12.5’ and 6’) without blur or pixelation of check edges which is typically observed in CRT or PDP VDUs.

### *Section 2—Electrophysiological measurements*

**Fig. 5:**
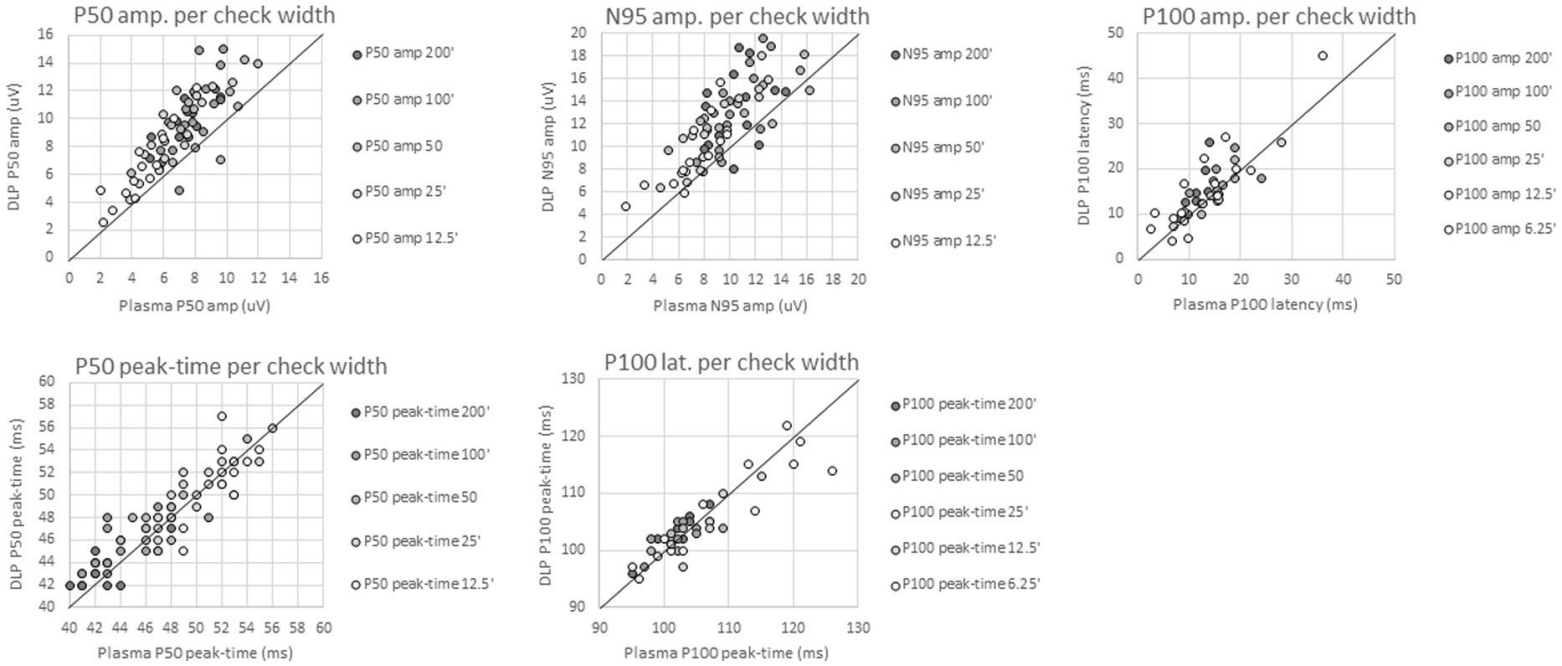
Scatterplots for main response components of the PERG and PVEP recorded from each device. The DLP values are plotted on the Y-axis and PDP values plotted on the X-axis. The diagonal line represents a *Y* = X fit whereby values falling along this line would indicate perfect agreement between devices

Simultaneous pattern VEP and PERGs were recorded to a range of check widths presented to the participant binocularly viewing the DLP laser projector or an existing laboratory PDP VDU. Response peak-times and amplitude were all significantly correlated (*p* =  < *0.05 all results),* showing strongly positive correlations relationships for all components, P50 amplitude (*r* = 0.841), P50 peak-time (*r* = 0.905), N95 amplitude (*r* = 0.766), P100 amplitude (*r* = 0.829) and P100 peak-time (*r* = 0.805) as observed in Table 1 and Fig. 5. Scatter plots of these data (Fig. 5) illustrate the high correlation between two devices. However, this demonstrated that DLP response amplitudes were linearly larger than PDP response amplitudes, represented as data points above than the total agreement line (*y* = *x*, Fig. 5). This appeared to be increase linearly with check width, so that the increase in amplitude was predictable with increased check width. This not observed for PERG or PVEP peak-time.

**Table 1:** Raw amplitude and peak-time measurements from the plasma display panel (PDP) and the digital light processing (DLP) device which were photometrically and spatially matched

Component measured	PDP (mean ± 1 SD)	DLP (mean ± 1 SD)	Difference Plasma (mean ± 1 SD)	Correlation (*r* =*)*	Signif. level (*p* =)
P50 amplitude (µV)	6.96 (± 2.11)	9.12 (± 2.85)	2.16 (± 1.57)	0.841	* < 0.000^1^
P50 peak-time (ms)	47.59 (± 4.51)	47.97 (± 3.92)	0.39 (± 1.92)	0.905	0.083^2^
N95 amplitude (µV)	9.51 (± 2.83)	12.11 (± 3.57)	2.60 (± 2.30)	0.766	* < 0.000^1^
P100 amplitude (µV)	13.79 (± 6.28)	15.60 (± 7.33)	1.80 (± 4.10)	0.829	*0.007^1^
P100 peak-time (ms)	105.52 (± 7.43)	105.90 (± 8.66)	0.38 (± 5.16)	0.805	0.873^2^

Amplitudes were normally distributed with peak-times non-normally distributed; ^1^Assessed with paired-samples *t*-test as data were normally distributed. ^2^Assessed using a related-samples Wilcoxon signed-rank test as data were non-normally distributed. * indicates a significant difference between devices at *p* < 0.05

This observation was further evidenced by significant group differences observed in P50 amplitude, N95 amplitude and P100 amplitude (Table 1). PERG P50 and N95 measures were larger from the DLP device by a mean of 2.16 µV and 2.60 µV, respectively. A less clinically significant, but statistically significantly, larger P100 component was observed from the DLP device, with the mean amplitude larger by 1.8 µV. P50 and P100 peak-times were not significantly different, with a mean difference of only 0.39 ms and 0.38 ms, respectively, although the P100 peak time appeared slightly earlier for smaller check widths from the DLP device than PDP device. These data are summarised in Table 1.

**Fig. 6:**
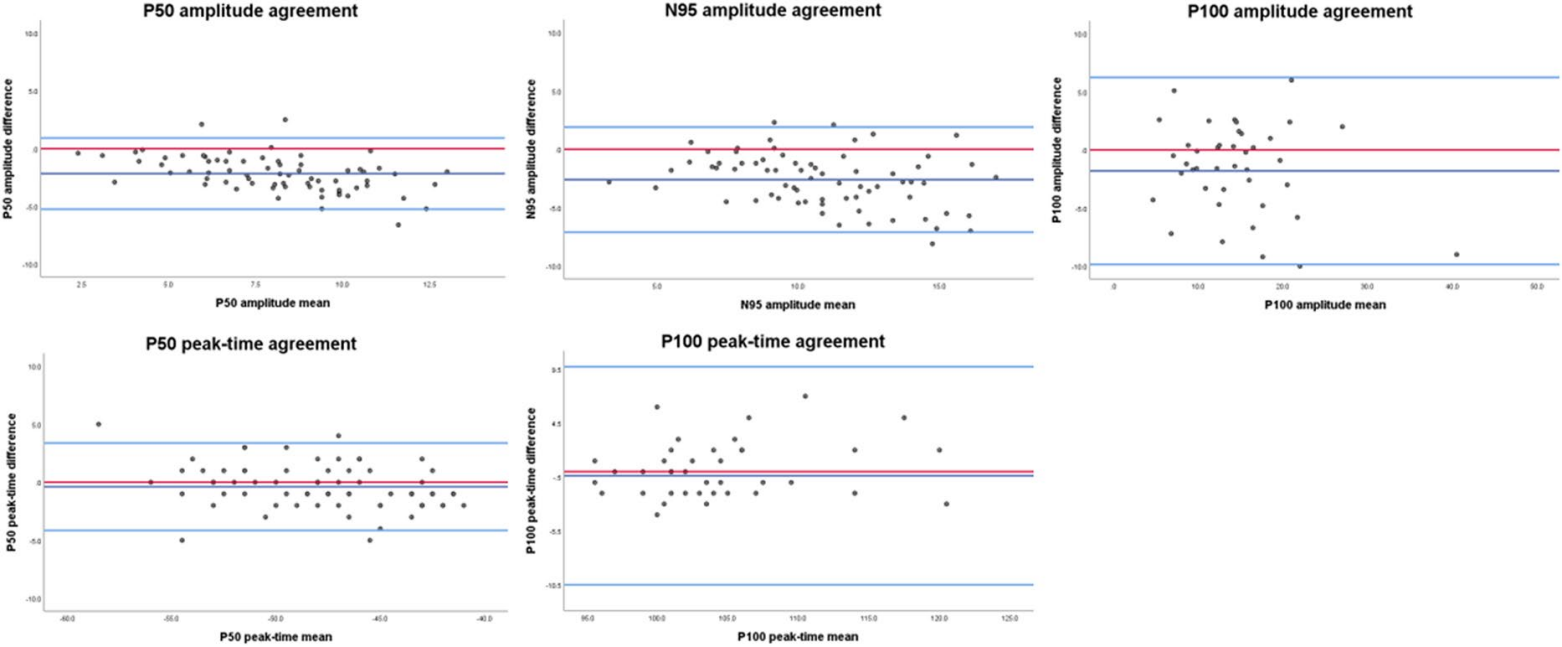
Bland–Altman plots for the Limits of Agreement (LoA) for PERG and PVEP differences between the two devices. The red line indicates zero (i.e. the closer to this line signifies better agreement. As can be seen, the mean (dark blue lines) for amplitude measurements differ significantly from zero for all components whereas peak-times are very close to zero, as consistent with other data. Importantly, there appears to be good distribution of data within the 95% confidence limits (light blue lines), suggesting that whilst skewed, there is high agreement between devices. This suggests systematic bias of data for amplitude

Bland–Altman plots were plotted for each respective major component for all check widths combined to assess the limits of agreement for the two devices (Fig. 6). This showed high agreement for all PERG and PVEP components, however for amplitude measurements a significant skew was observed for the mean from zero as expected from our scatterplots. These data suggest that there is high agreement between devices, but there is a systematic bias of data whereby the mean amplitude is larger from the DLP device than the PDP device, consistent with our other findings.

## Discussion

This study aimed to examine DLP laser projectors as potential VDUs for routine pattern use in visual electrophysiology tests. Our findings confirm that the Viewsonic DLP laser projector tested in this study is very suitable for these purposes, providing high luminance, high contrast and fast temporal profiles required of visual stimulators. Importantly the patterns are produced with temporally identical and balanced luminance on (rise) and off (fall) timings. Furthermore, we demonstrate that physiological responses recorded from the tested device is similar to those from existing, established VDUs at our centre. The tested DLP laser projector produced responses of comparable peak-times to existing validated systems, though response amplitudes were larger from the DLP device. The confirmation that some DLP laser projectors are suitable for electrophysiology testing is particularly important at the time of writing, given the increasing difficulty in sourcing suitable reliable VDUs and decreasing availability of remaining obsolete devices.

To our knowledge, the reported literature evaluating DLP technology in this setting is very limited with only one study assessing its use for chromatic VEPs [11]. Alternative solutions such as LCD VDUs have been well documented, but highlight their significant limitations in terms of transient luminance artefacts and input lag [4–6, 12–14]. Whilst CRT and PDP technology is robust and suitable for use, these devices are now obsolete and modern solutions are required. Although some OLED displays show useful properties as VDUs [13, 15], it is the authors experience that a proportion of these devices exhibit an input lag jitter, similar to that observed with the HiSense device in this study and are therefore unsuitable for use. The main DLP device assessed in this study appears to be a robust, fast and capable stimulator for visual electrophysiology testing.

Whilst we found that the assessed DLP laser projector is highly suitable for clinical testing, the individual model specification is evidently critical. We discovered a detrimental input lag and jitter in the second Hisence DLP laser projector device, which prevented any further appraisal of this device. It is likely that this jitter was caused by resampling the incoming signal within the projector system, which caused a frame shift or desynchronisation of the resultant signal output. All digital processing settings within the projector had been turned off for testing, but it is possible that some devices retain an inherent processing of incoming signals which makes them unsuitable for electrophysiology testing. The authors personal observations are that some OLED devices suffer a similar input lag jitter, but this is similarly model dependent. Of note, the Hisense device was advertised as an ‘entertainment’-based DLP projection system, whereas the Viewsonic device was advertised as an office/work-based projector. It is possible that entertainment-based DLP devices may process video signals to enhance performance, which evidently may preclude their use for visual electrophysiology. Based on these observations, we strongly recommend that anyone considering use of DLP projectors should assess individual model feasibility before clinical implementation.

We found response times to be very fast for the DLP device, with rise and fall times of 0.5–1 ms. This is comparable or faster than that observed for CRT and LCD stimulators, respectively [16]. The manufacturer data for the DMD chips (Texas Instruments) suggest that response times may far exceed that recorded in this study (movement speeds up to 10,000 Hz), suggesting that these response times may reflect a simplification or limitation of the graphical output from the Espion E^3^ system [7]. During set up, a 60 Hz calibration file was generated, this could theoretically be increased to 100 Hz with this system but comes at the compromise of resolution due to the pixel clock rate. Overall, the DLP’s luminance and contrast ratios were widely sufficient for clinical testing and far exceeded the minimum standards required for PERG and PVEP testing [2, 3]. Importantly, we found a significant input lag for the DLP device, taking 50 ms from trigger to stimulus change. Whilst significant, this was very stable and adjustments can be easily made for this input lag by adjusting time zero to coincide with the onset or half-point of reversal change, as indicated in clinical standards [2, 3].

We found that warm-up time (i.e. time from a ‘cold start’ turn on to being fully operational) was immediate for the PDP device, but took around two minutes for the Viewsonic DLP device. This time is certainly an acceptable level for clinical circumstances, particularly since LCD and CRT VDUs can take or exceed a 60 min warm-up [12, 17], after this LCDs are also sensitive to changes in ambient temperature [12]. Furthermore, we found no delay in response time over this period, suggesting the device is fully operational within two minutes warm-up. This is in further contrast to LCD devices which have slow response time warm-up periods, with some devices taking up to one hour until reaching optimal response time [18]. Certainly, based on the mechanical properties of stimulus presentation which is based on the DMD chip speed, we would not have expected any delay in response time over this period.

Luminance properties of the device were very advantageous, capable of maintaining high contrast at mean luminance around 300 cd/m^2^. We found mild luminance variance across the projection screen, but this was within 91.8% of maximum so within acceptable recording standards [2, 3]. Nevertheless, the luminance distribution appeared to follow a pattern whereby those closest to the DLP laser projector had higher luminance than those furthest away. This may be a feature of the ultra-short throw ratio used of this projector, creating highly oblique angles to the projection screen. It is suspected that DLP laser projectors with longer throw ratios may show less of this spatial variance in luminance.

We found that the spectral properties of the stimulus showed a large peak in the ‘blue’ wavelength with broader energy at longer wavelengths (Fig. 1D). There is no specific reference to the spectral properties of stimuli for clinical PERGs or PVEPs [2, 3]. Existing visual stimulators have widely varying spectral properties, so the spectral profile observed for the DLP device is likely insignificant in this context, as the correlated colour temperature was very close to 6500K (6495K). Furthermore, the spectral properties of DLP laser projectors may vary per device depending on the composition of the colour wheel. Perhaps most curious of our observations was the perceived ‘rainbow effect’, which occurred with fast eye movements (supplementary Fig. 2). This is a type of stroboscopic artefact giving a spectral inhomogeneity of the pattern stimulus due to the colour wheel used, and is particularly marked for white checks. It is a result of the colour being rendered sequentially through the colour wheel causing temporal inhomogeneities, typically at 2–4 times the framerate. From our observations, this was only apparent with very rapid eye movements and perception varied according to observer. Nevertheless, the influence of this finding on patients with unstable fixation (i.e. children) or involuntary eye movements (i.e. nystagmus) is uncertain.

We suspect that the rainbow effect would have little significant influence on the PERG or PVEP, as the colour wheel frequency was measured here to be 120 Hz (twice the 60 Hz framerate) which is far faster than the time-locked presented visual stimuli, temporal resolution of visual contrast systems [19] and is around the temporal resolution limit of cone photocurrents [20]. Furthermore these artefacts are not constant inhomogeneities, instead are rapidly changing temporally, so any resultant physiological differences would likely average to noise levels. Nevertheless, this may be dependent on device used, as some newer or more expensive DLP devices may use × 4 or higher colour wheel frequencies relative to framerate, which would minimise this effect. Early DLP projection devices used a 1 × colour wheel making the rainbow effect markedly evident for most observers, but are now rarely used. Furthermore, technical developments in this area are continuing and are likely to further minimise this effect, such as development of the 3-chip DLP (comprising of three DMD chips for red, green and blue lasers) or three laser DLPs (removing the need for the colour wheel). Considering these points, this effect is considered to have a negligible influence on the PERG or PVEP.

In the physiological experiments, we did not find any significant differences in peak-time between P50 and P100 components between devices, suggesting the tested DLP device performed similarly to our existing systems in this respect. The largest discrepancy in our results were larger responses for PERGs and PVEPs and earlier peak-times for small check width PVEPs from the DLP laser projector than the existing PDP VDU, despite spatial and photometric matching. This is an interesting finding, which suggests that the DLP device may perform better than existing PDP devices. The explanation for this difference in view of photometric and spatial matching may, we suspect, result from originate from two possible mechanisms. Firstly response times from the DLP device are faster than PDP VDUs, or secondly the improved resolution of DLP laser projection systems which affects different or enhanced physiological properties.

Response times observed from the DLP stimulator assessed in this study were fast, in the order of 0.5–1 ms. It is possible, that this faster response time of DLP relative to existing PDP VDUs may allow better temporal synchronisation of the physiological substrate of interest. An abrupt response change may theoretically cause more simultaneous activation of retinal and neural cells which may therefore improve response amplitude as observed in this study. It has been demonstrated that response times do not significantly alter the PVEP below 10 ms, although likely alter between 8 and 16 ms to affect the PVEP which may explain our findings [21]. This is supported by the upper limit of frequency–response curves of the pattern VEP being 15-20 Hz [22]. Therefore, faster rise times are a likely cause for the larger PERG and PVEP amplitudes observed in our study, which is likely advantageous for clinical testing but highlights a need for locally derived reference data for implementation of these new devices.

It is fairly well known that whilst CRT and PDP systems are suitable VDUs, they have relatively poor spatial resolution due to pixel size and therefore edge contrast can be low. Reducing edge contrast can have a direct effect on the PVEP amplitude, as the pattern stimulus waveform becomes more sinusoidal similar to a change in modulation transfer function [23, 24]. Therefore, the relative higher resolution and sharpness of a DLP stimulus may therefore improve the respective retinal contrast, which would be particularly evident to small check widths as observed in PERG and PVEP data of our study. We suspect these changes may, at least in part, be responsible for the differences in amplitude between devices observed.

A very beneficial feature of DLP laser projectors is that they are capable of extra-large field stimulation within ultra-short throw ratios, meaning very large field sizes can be achieved without the need for large laboratory space. We calculate that the Viewsonic projector as used in this study, at a working distance of 125 cm, could present stimuli in visual fields of up to 90 degrees. Whilst large field sizes are particularly useful for paediatric practice, there comes a point whereby larger field size becomes detrimental to the PVEP P100 component. With increasingly large field sizes the paramacular PVEP components become more pronounced, and if large enough they can degrade the macular driven P100 component of interest [10]. There may be some applications for large field sizes which are beneficial to avoid short viewing distances, such as for the mfERG or mfVEP, but for routine clinical PERG and PVEP testing, it seems that exceeding a 30 degree field may not yield any significant benefit, hence our aim to spatially match existing PDP system dimensions.

Lastly, whilst our study assessed the DLP device in front-projection mode, it is likely that in clinical circumstances a back-projection would be far more beneficial to avoid any potential interference of the projection beam by patients, staff or equipment.

## Conclusion

In conclusion, we demonstrate that the DLP laser projector assessed in this study is a suitable VDU for use in visual electrophysiology testing. This DLP laser projector was easily used with commercially available visual electrophysiology systems and provided stimuli compatible with ISCEV standards for the PERG and PVEP. We observe similar PVEP and PERG values compared to an already established VDU, with some amplitude values better than existing systems. For other centres considering DLP laser projection systems, it is important to carefully appraise the manufacturer specifications and model of each DLP device to avoid one which suffers from the detrimental jitter observed in one of the devices tested in this study. Future research is needed to assess the test–retest repeatability of PERGs and PVEPs recorded to a DLP stimulus, alongside photometric measurements over long time periods to assess for any age-related changes in stimulus parameters.

## Supplementary Information

Below is the link to the electronic supplementary material.

Supplementary file1 (DOCX 1302 kb)

## Abbreviations

DLP: Digital light processing
DMD: Digital micromirror device
PDP: Plasma display panel
PERG: Pattern electroretinogram
PVEP: Pattern visual evoked potential

## References

[1] Hoffmann MB, Bach M, Kondo M (2021). ISCEV standard for clinical multifocal electroretinography (mfERG) (2021 update). Doc Ophthalmol.

[2] Bach M, Brigell MG, Hawlina M, et al (2013) ISCEV standard for clinical pattern electroretinography (PERG): 2012 update. 1–7. 10.1007/s10633-012-9353-y 10.1007/s10633-012-9353-y 23073702

[3] Odom JV, Bach M, Brigell M (2016). ISCEV standard for clinical visual evoked potentials: (2016 update). Doc Ophthalmol.

[4] Ghodrati M, Morris AP, Price NSC (2015). The (un)suitability of modern liquid crystal displays (LCDs) for vision research. Front Psychol.

[5] Matsumoto CS, Shinoda K, Matsumoto H (2014). Comparisons of pattern visually evoked potentials elicited by different response time liquid crystal display screens. Ophthalmic Res.

[6] Matsumoto CS, Shinoda K, Matsumoto H (2013). Liquid crystal display screens as stimulators for visually evoked potentials: flash effect due to delay in luminance changes. Doc Ophthalmol.

[7] Plano T (2008) The Digital Micromirror Device A Historic Mechanical Engineering Landmark

[8] Brigell M, Bach M, Barber C (2003). Guidelines for calibration of stimulus and recording parameters used in clinical electrophysiology of vision. Doc Ophthalmol.

[9] Kappal SJ, Narasimhan SG (2010) Illustrating motion through DLP photography. 9–16. 10.1109/CVPRW.2009.5204315

[10] Shawkat FS, Kriss A (1997). Effects of experimental scotomata on sequential pattern-onset, pattern-reversal and pattern-offset visual evoked potentials. Doc Ophthalmol.

[11] Dussan Molinos L, Huchzermeyer C, Lämmer R (2022). Blue-yellow VEP with projector-stimulation in Glaucoma. Graefes Arch Clin Exp Ophthalmol.

[12] Fox M, Barber C, Keating D, Perkins A (2014). Comparison of cathode ray tube and liquid crystal display stimulators for use in multifocal VEP. Doc Ophthalmol.

[13] Matsumoto CS, Shinoda K, Matsumoto H (2014). What monitor can replace the cathode-ray tube for visual stimulation to elicit multifocal electroretinograms?. J Vis.

[14] Nagy BV, Gémesi S, Heller D (2011). Comparison of pattern VEP results acquired using CRT and TFT stimulators in the clinical practice. Doc Ophthalmol.

[15] Cooper EA, Jiang H, Vildavski V (2013). Assessment of OLED displays for vision research. J Vis.

[16] Elze T (2010). Achieving precise display timing in visual neuroscience experiments. J Neurosci Methods.

[17] Metha AB, Vingrys AJ, Badcock DR (1993). Calibration of a color monitor for visual psychophysics. Behav Res Methods Instrum Comput.

[18] Liang H, Badano A (2007). Temporal response of medical liquid crystal displays. Med Phys.

[19] Donner K (2021). Temporal vision: measures, mechanisms and meaning. J Exp Biol.

[20] van Hateren JH, Lamb TD (2006). The photocurrent response of human cones in fast and monophasic. BMC Neurosci.

[21] Barber C (1981). Inherent characteristics of visual stimulus systems and their effect on the visual evoked potential. Clin Phys Physiol Meas.

[22] Regan D (1975). Recent advances in electrical recording from the human brain. Nature.

[23] Adachi-Usami E (1981). Human visual system modulation transfer function measured by evoked potentials. Neurosci Lett.

[24] Marcar VL, Wolf M (2021). An investigation into the relationship between stimulus property, neural response and its manifestation in the visual evoked potential involving retinal resolution. Eur J Neurosci.

