# Supplementary material for: Assessment of digital light processing (DLP) projector stimulators for visual electrophysiology
Source: Doc Ophthalmol. 2023 Jan 8;146(2):151–63. doi: 10.1007/s10633-022-09917-4 (PMC10082110; doi:10.1007/s10633-022-09917-4)
Supplement: Supplementary file 1 — Supplementary file1 (DOCX 1302 kb) [file 10633_2022_9917_MOESM1_ESM.docx]

# Supplementary figures


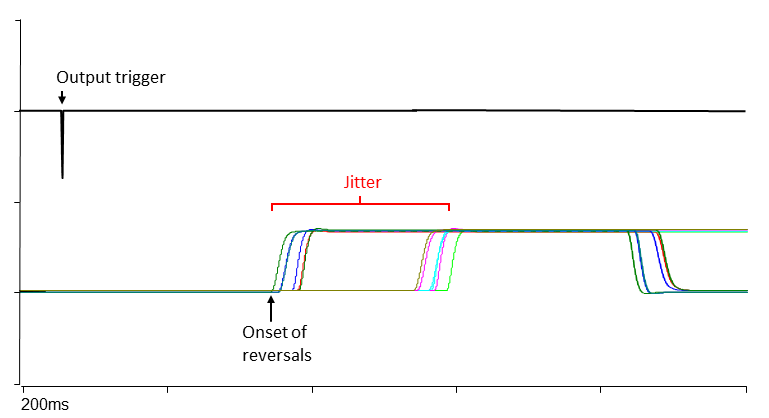


***Supplementary figure 1 – Temporal jitter of reversal stimuli with the Hisense DLP laser projector.*** *The output trigger (2ms), top black trace is maintained constant. The bottom coloured traces are the signal from the photodiode recording a check reversing. As can be observed, the onset of reversal varies widely with some onset jitter exceeding 200ms.*

**
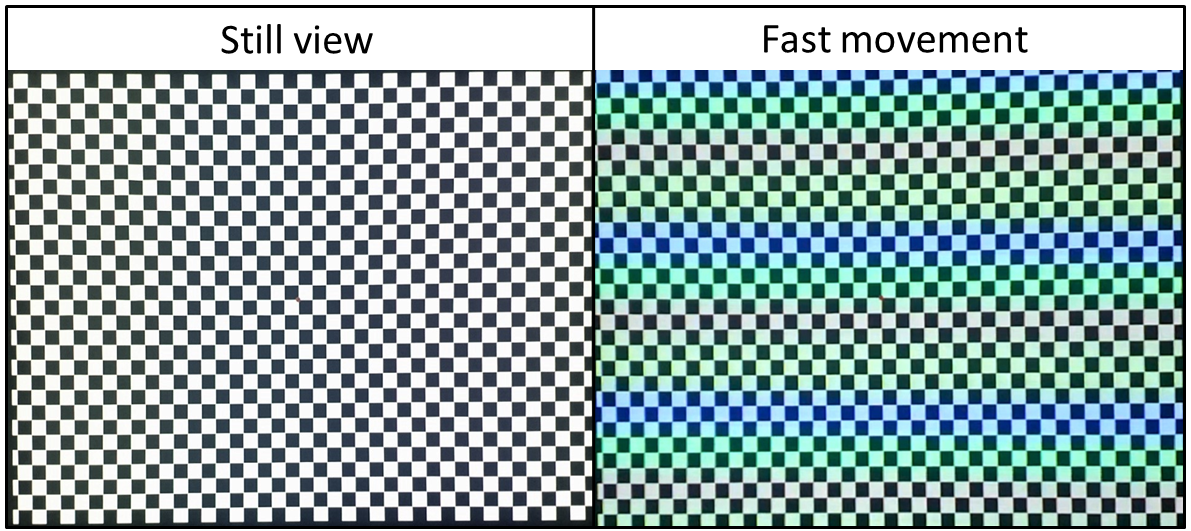
**

***Supplementary figure 2 – demonstration of the DLP rainbow effect.*** *The ‘still view’ is a static picture taken from the visual stimulus used in this study, as can be observed, there is no heterogenicity in the colour of white checks. The ‘fast movement’ is a still image taken from a video whereby the camera was shaken from side to side to demonstrate the ‘rainbow effect’ whereby there is heterogenicity of colours of white checks.*
